# Retracted: Galectin-1, -4, and -7 Were Associated with High Activity of Disease in Patients with Rheumatoid Arthritis

**DOI:** 10.1155/2020/8364502

**Published:** 2020-11-28

**Authors:** Autoimmune Diseases

In the article titled “Galectin-1, -4, and -7 Were Associated with High Activity of Disease in Patients with Rheumatoid Arthritis” [1], the authors have identified that an incorrect dataset was used during the analysis. After repeating the analysis with the correct data, the conclusions of the article are no longer valid. The article is, therefore, being retracted with the agreement of the authors and our editorial board. The authors apologize for this error.
